# The Use and Limitations of Exome Capture to Detect Novel Variation in the Hexaploid Wheat Genome

**DOI:** 10.3389/fpls.2022.841855

**Published:** 2022-04-12

**Authors:** Amanda J. Burridge, Mark O. Winfield, Paul A. Wilkinson, Alexandra M. Przewieslik-Allen, Keith J. Edwards, Gary L. A. Barker

**Affiliations:** ^1^School of Life Sciences, University of Bristol, Bristol, United Kingdom; ^2^Institute of Systems, Molecular and Integrative Biology, University of Liverpool, Liverpool, United Kingdom

**Keywords:** wheat, *Triticum aestivum*, introgression, exome capture, exome capture sequencing, sequence variation

## Abstract

The bread wheat (*Triticum aestivum*) pangenome is a patchwork of variable regions, including translocations and introgressions from progenitors and wild relatives. Although a large number of these have been documented, it is likely that many more remain unknown. To map these variable regions and make them more traceable in breeding programs, wheat accessions need to be genotyped or sequenced. The wheat genome is large and complex and consequently, sequencing efforts are often targeted through exome capture. In this study, we employed exome capture prior to sequencing 12 wheat varieties; 10 elite *T. aestivum* cultivars and two *T. aestivum* landrace accessions. Sequence coverage across chromosomes was greater toward distal regions of chromosome arms and lower in centromeric regions, reflecting the capture probe distribution which itself is determined by the known telomere to centromere gene gradient. Superimposed on this general pattern, numerous drops in sequence coverage were observed. Several of these corresponded with reported introgressions. Other drops in coverage could not be readily explained and may point to introgressions that have not, to date, been documented.

## Introduction

The bread wheat (*Triticum aestivum*) pangenome is a patchwork containing translocations and introgressions from wheat’s wild relatives (Przewieslik-Allen et al., 2021) as well as numerous deletions. Some of these features may be present in only a handful of accessions coming from a limited geographic area whilst others may be prevalent and present in varying combinations across many accessions. Some variable regions may have occurred naturally by mutation or as a consequence of promiscuous pollination events between wheat and one of its primary relatives (He et al., 2019). Others are the result of breeding efforts (Schneider et al., 2008) using traditional methods to introduce segments from progenitors and close relatives or, more recently, using more advanced methods to perform wide crosses (Cseh et al., 2019; Devi et al., 2019; King et al., 2019; Xu et al., 2020). Regardless of their origin, the number of these variable regions that have been documented is probably not a genuine reflection of their true number; breeding companies may not have reported, and indeed may not know, all the introgressed regions in their elite lines, and chance events in landrace accessions are unlikely to have been documented at all. It would seem highly likely, therefore, that there are numerous unknown, introgressions present in modern wheat accessions (Przewieslik-Allen et al., 2021).

With this in mind, and with modern techniques allowing for wide crossing with increasing success, increasingly diverse wheat accessions are becoming available for pre-breeding (Hao et al., 2020). To be of use to research and breeding programs, such material needs to be tracked using either targeted molecular markers (Singh et al., 2018; Rasheed and Xia, 2019) or sequencing. The former has most frequently been used because it offers low cost and high throughput (Zhang J. et al., 2017; Zhang W. et al., 2017; Przewieslik-Allen et al., 2019). However, marker probes will only hybridize to, and so provide a signal for, the sequences for which they were designed. Thus, wheat genotyping markers intended for introgression detection need to be designed using sequences from a combination of wheat and the progenitors and relatives thought to have been the source of those introgressions (Wang et al., 2014; Zhang J. et al., 2017; Przewieslik-Allen et al., 2019). Where the source of introgressed material is unknown, and so not included in probe design, genotyping is unlikely to track such regions.

Sequencing, having no requirement for prior knowledge of the target, does not suffer from such a problem. However, the size and complexity of the wheat genome create problems in this regard. *T. aestivum* has a large (∼17 Gb) polyploid and highly repetitive genome of which the exome constitutes less than 5% (International Wheat Genome Sequencing Consortium [IWGSC], 2014). To sidestep these issues, targeted sequencing approaches, such as exome capture, are used (Kaur and Gaikwad, 2017). In wheat, several exome capture systems that incorporate capture probe sets derived from both hexaploid wheat and its relatives have been proposed (Winfield et al., 2012; Gardiner et al., 2019; He et al., 2019). The capture probes themselves can tolerate some degree of mismatch thus allowing the capture of sequences outside the immediate confines of the species from which they are derived. The Roche SeqCap EZ system can tolerate up to 10% (Roche pers com) and the Arbor Biosciences myBaits system can tolerate up to 20% divergence from the target sequence (Arbor Biosciences, 2021). This property is highly beneficial where the exact source of the material is unknown and has been exploited to capture sequences from diverse origins in the wild relative species of cotton (Salmon et al., 2012), cows (Cosart et al., 2011), and humans (Jin et al., 2012) as well as in wheat (Saintenac et al., 2011; Henry et al., 2014; He et al., 2019).

We recently described the variable sequence coverage of the wheat variety ‘Player’ when exome capture data were aligned to the ‘Chinese Spring’ reference sequence (Przewieslik-Allen et al., 2021). Distinct drops in sequence coverage were evident in chromosomes 2A and 2B which correlated with introgressions from *Aegilops ventricosa* and *Triticum timopheevii*, respectively. As the use of exome capture prior to sequencing followed by alignment to a standard reference is common practice, the potential for this to be disrupted by introgressions is a concern, especially as many interesting, rare, and novel alleles may be located in regions derived from wild relatives. This was investigated using 10 elite *T. aestivum* cultivars and 2 *T. aestivum* landrace accessions used in breeding.

## Results

### Sequence Coverage

Using gene and promoter sequence capture (Gardiner et al., 2019), 12 *T. aestivum* accessions (10 elite varieties and 2 landrace accessions) were sequenced and total coverage compared. Total reads were between 48,255,718 and 145,897,760 per accession; after quality trimming and alignment to the IWGSC RefSeq v1.0 ‘Chinese Spring’ reference (International Wheat Genome Sequencing Consortium [IWGSC], 2018), there were between 20,973,857 and 63,662,179 uniquely mapped, paired reads per accession (Table 1).

**TABLE 1:** Read statistics before and after trimming with alignment statistics for total mapped and uniquely mapped reads.

Variety	Total reads	Trimmed reads	Total mapped paired reads	Uniquely mapped paired reads
Apogee	66,890,848	64,719,732 (96.8%)	33,445,424	28,796,984 (86.1%)
Bacanora	80,558,016	77,878,454 (96.7%)	38,939,227	33,411,894 (85.8%)
Bobwhite	57,245,246	55,371,124 (96.7%)	27,685,562	24,139,596 (87.2%)
Boregar	48,255,718	46,603,262 (96.6%)	24,127,859	20,973,857 (86.9%)
Cadenza	72,469,144	70,237,432 (96.9%)	36,234,572	31,033,245 (85.6%)
KWS Kielder	65,891,042	63,791,268 (96.8%)	32,945,521	28,236,689 (85.7%)
Maris Huntsman	52,673,928	50,665,506 (96.2%)	25,332,753	21,992,928 (86.8%)
Pavon 76	145,897,760	141,207,878 (96.8%)	72,948,880	63,662,179 (87.3%)
Renan	56,921,314	54,670,680 (96.0%)	28,460,657	24,854,489 (87.3%)
Riband	68,965,154	66,392,980 (96.3%)	33,196,490	28,910,396 (87.0%)
Watkins 141	51,300,352	49,480,812 (96.5%)	24,740,406	21,238,652 (85.8%)
Watkins 777	99,274,852	95,527,090 (96.2%)	49,637,426	43,055,487 (86.7%)

**FIGURE 1:**
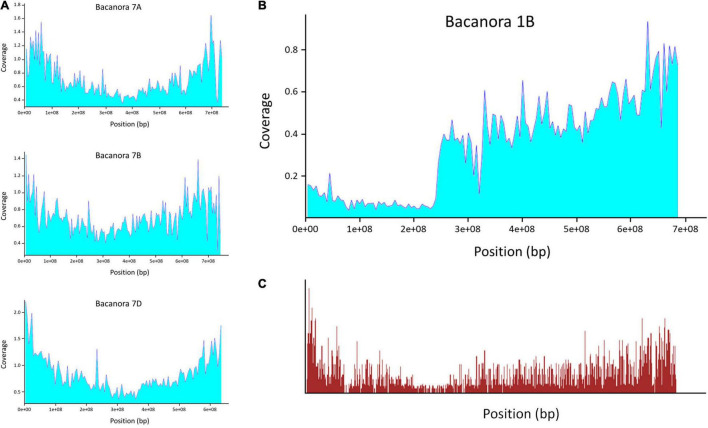
Read coverage for the accession ‘Bacanora’ after alignment to IWGSC ‘Chinese Spring’ assembly version 1.0. **(A)** Read coverage across chromosomes tended to be higher toward the telomeres and lower across the centromere. **(B)** Chromosome 1B shows a clear drop in coverage across the short arm (NB in all plots, chromosome short arms are on the left). **(C)** Location and density of capture probes across chromosome 1B (data from Gardiner et al., 2019).

Sequence coverage across chromosomes displayed a characteristic pattern; that is, there was a greater depth of coverage toward the ends of chromosome arms and lower coverage across centromeres (Figure 1A). However, this overall pattern was, in places, interrupted by regions of pronounced reduction in sequence coverage. These regions were not seen on all chromosomes or simultaneously in all accessions (Supplementary File 1). The most pronounced of these reductions in coverage was observed in ‘Bacanora’, ‘Bobwhite’, and ‘KWS Kielder’ and extended across the whole of the short arm of chromosome 1B (c. 240 Mb; Figure 1B) in line with the well documented and prevalent 1RS/1BL *Secale cereale* translocation (Rabinovich, 1998). There was no reduction in capture probe density across 1BS (Figure 1C) and the nine accessions without the 1RS translocation do not show a reduction in read coverage across this chromosome arm (Supplementary File 1: 1B).

**FIGURE 2:**
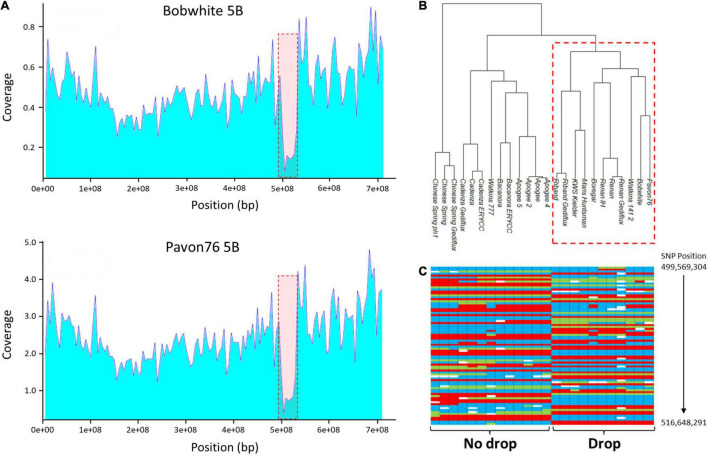
**(A)** Average depth of coverage for chromosome 5B in the accessions ‘Bobwhite’ and ‘Pavon 76’; both show a drop in read coverage on the long arm at approximately position 490,000,000–540,000,000. **(B)** Dendrogram based on the 1,749 Axiom markers mapped to chromosome 5B; the 8 varieties (‘Bobwhite’, ‘Boregar’, ‘KWS Kielder’, ‘Maris Huntsman’, ‘Pavon 76’, ‘Renan’, ‘Riband’, and ‘Watkins 141’) with the drop in read coverage cluster. **(C)** A sample of the SNP calls across the interval 499,569, 304–534,345,241 highlighting the difference between the two groups (blue and red are the alternative homozygote calls; green indicates heterozygote calls).

**FIGURE 3:**
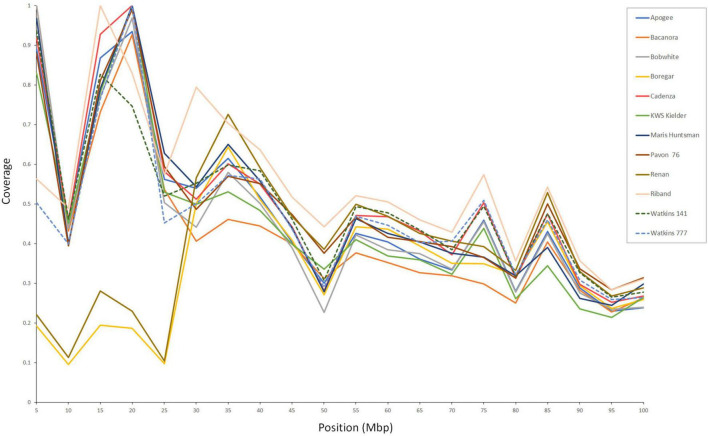
Sequence coverage across the first 100 Mb of chromosome 2AS. The two accessions, ‘Boregar’ and ‘Renan’, show reduced coverage across the first 25–30 Mb which corresponds with the size of the known introgression from *Ae. ventricosa* (Robert et al., 1999).

Other large drops in coverage were seen on 2BL, 2DL, and 5BL (Figure 2) which extended over approximately 85, 45, and 40 Mb, respectively. Additional, smaller drops in coverage were also observed in telomeric regions, such as 2AS (Figure 3), 7DL, and an additional region in 2DL (Supplementary File 1).

### Cluster Analysis of Accessions

To determine whether there was any relationship between the lines that shared read coverage profiles, cluster analysis was performed with Axiom 35K Wheat Breeders’ Array genotyping data (Allen et al., 2016). Analysis was performed on markers specific to the chromosomes 2B (2,083 markers), 2D (2,237 markers), and 5B (1,749 markers). Accessions showed a pattern of clustering that corresponded with the drops in coverage (Figure 2B and Supplementary File 2). For chromosome 5B, for example, the 12 accessions separated into two main clusters; the accessions thought to contain the deletion fell into one cluster while those with even sequence coverage fell into the other. The separation into two clusters was driven by the markers spanning the drop. Across the interval corresponding to the decline in read coverage on chromosome 5B (position 499,569,304–534,345,241), there were 141 single nucleotide polymorphism (SNP) markers; for these markers, the mean percentage similarity between the genotype calls for ‘Chinese Spring’ and those of the eight accessions displaying the drop in coverage was only 13.3%. This compares to a mean similarity of 59.1% for the SNP calls across the rest of the chromosome (Figure 2C).

### Bibliographic Search for Introgressions

A number of wheat introgressions reported in the literature were assembled (Table 2) to determine whether there was any relationship between them and the patterns of reduced sequence coverage observed in this study. The large drop in coverage on 1BS, for example, is present in those varieties (Bacanora, Bobwhite, and KWS Kielder) known to possess a whole arm translocation from *S. cereale*; we have previously reported this ourselves based on genotyping results using the Axiom High-Density Array (Winfield et al., 2015). Other chromosomal regions with reduced read coverage were also related to regions of known introgressions. However, not all the reports of introgressions that we found in the literature had a corresponding drop in sequence coverage, and in some cases, there was a drop in sequence coverage for which no source was found. Notable deletions, such as that on 1DL of ‘Cadenza’, highlight the similarity between deletions and introgressions in sequence coverage.

**TABLE 2:** Introgressions and deletions reported in the literature for the accessions in this study.

Cultivar	Gene	Chromosome	Source	References
Bacanora		1BS	*S. cereale*	Driever et al., 2014
	*Ppd1*	2DS		Driever et al., 2014
Bobwhite		1BS	*S. cereale*	Warburton et al., 2002
	*Rht8a*	2DS		Worland et al., 1998
Boregar	*Pch1*	7DL	*Ae. ventricosa*	Burt and Nicholson, 2011
Cadenza	*Eps*	1DL	Deletion	Zikhali et al., 2016
	*Yr7*	2BL	*T. durum*	Marchal et al., 2018
	*Yr5*	2BL	*T. spelta*	Marchal et al., 2018
	*Sbm1*	5DL		Kanyuka et al., 2004
	*Yr6*	7BS	*T. aestivum*	Ma et al., 2015
KWS Kielder		1BS	*S. cereale*	Przewieslik-Allen et al., 2021
Maris Huntsman	*Yr3a*	1B	*T. aestivum*	Bai et al., 2014
	*Pm6*	2BL	*T. timopheevii*	Wang et al., 2005
	*Yr13*	2BS	*T. aestivum*	Bai et al., 2014
	*Lr13*	2BS	*T. aestivum*	McIntosh et al., 1995
	*Yr34*	5AL	*T. monococcum*	Chen et al., 2021
	*Yr4a*	6B	*T. aestivum*	Bai et al., 2014
	*Yr2*	7BL	*T. aestivum*	Bai et al., 2014
	*Pm2*	5DS	*Ae. tauschii*	Wang et al., 2005
Pavon 76	*Lr10*	1A	*T. aestivum*	Singh and Rajaram, 1991
	*Yr29*	1BL	*T. aestivum*	Cobo et al., 2019
	*Lr46*	1BL	*T. aestivum*	Singh and Rajaram, 1991
	*Yr29*	1BL	*T. aestivum*	William et al., 2003
	*Yr7*	2BL	*T. durum*	Durbin et al., 1989
	*Lr13*	2BS	*T. aestivum*	Singh and Rajaram, 1991
	*Yr30*	3BS	*T. aestivum*	Boyd, 2005
	*Sr2*	3BS	*T. dicoccum*	Mago et al., 2014
	*Lr1*	5D	*T. aestivum*	Singh and Rajaram, 1991
	*Yr6*	7BS	*T. aestivum*	Wellings, 1986
Renan	*Pm4b*	2AL	*T. turgidum*	Chantret et al., 1999
	*Yr17*	2AS	*Ae. ventricosa*	Robert et al., 1999
	*Ppd-B1b*	2B		Kiseleva et al., 2007
	*Pch1*	7DL	*Ae. ventricosa*	Burt and Nicholson, 2011
Riband	*Lr17b*	2AS	*T. aestivum*	Pathan and Park, 2006
	*Stb15*	6AS		Arraiano et al., 2007
	*Pm4b*	2AL	*T. turgidum*	United Kingdom Cereal Pathogen Virulence Survey [UKCVS], 2004
	*Pm6*	2BL	*T. timopheevii*	United Kingdom Cereal Pathogen Virulence Survey [UKCVS], 2004
	*Pm2*	5DS	*Ae. tauschii*	United Kingdom Cereal Pathogen Virulence Survey [UKCVS], 2004

### Efficacy of Sequence Capture

**FIGURE 4:**
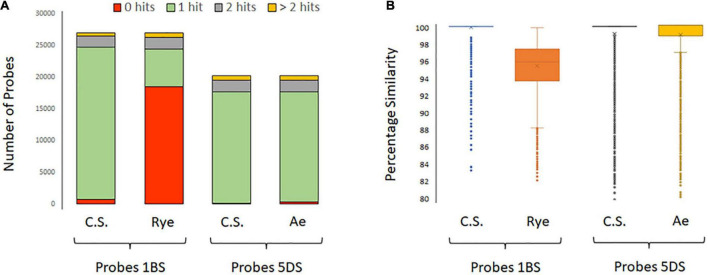
**(A)** Bar graphs showing the number of capture probes that had BLAST hits to ‘Chinese Spring’ chromosome 1BS (IWGSC v1), *S. cereale* chromosome 1RS (JADQCU000000000 v1 of the cultivar Weining), ‘Chinese Spring’ chromosome 5DS (IWGSC v1), and *Ae. tauschii* chromosome 5DS (PRJNA341983 assembly of *Ae. tauschii* subsp. *strangulata*). The number of probe sequences for chromosomes 1BS and 5DS was 26,985 and 20,253, respectively. The number of probes that produced a hit was 26,222 to ‘Chinese Spring’ 1BS, 8,419 to *S. cereale* 1RS, 20,082 to ‘Chinese Spring’ 5DS, and 19,872 to *Ae. tauschii* 5DS. There were more hits than probe sequences as some probes had multiple hits. **(B)** Box and whisker plots showing the percentage similarity between the probe sequences and their respective targets.

The accessions containing the 1RS.1BL translocation (‘Bacanora’, ‘Bobwhite’, and ‘KWS Kielder’) displayed a clear drop in read coverage across the short arm of 1B; we hypothesized that this was due to capture efficacy in the different backgrounds. The potential efficacy of probes to capture sequences from either ‘Chinese Spring’ or *S. cereale* was assessed by BLASTing their sequences to their respective assemblies. Capture probe sequences for chromosome 1BS (26,985 sequences) were BLASTed against the 1B pseudomolecule of ‘Chinese Spring’ and 1R of *S. cereale*. This resulted in 29,652 hits to ‘Chinese Spring’ 1BS and 12,120 hits to *S. cereale* 1RS. To both assemblies, some probes had multiple hits. The number of probe sequences that had a hit was 26,222 and 8,419, respectively. Those with a single hit were 23,969 and 5,822, respectively (Figure 4A), and the percentage similarity between probe sequences and their target was 99.8 and 95.6%, respectively (Figure 4B). That is, a greater number of probes matched the ‘Chinese Spring’ sequence and with greater percentage similarity.

**FIGURE 5:**
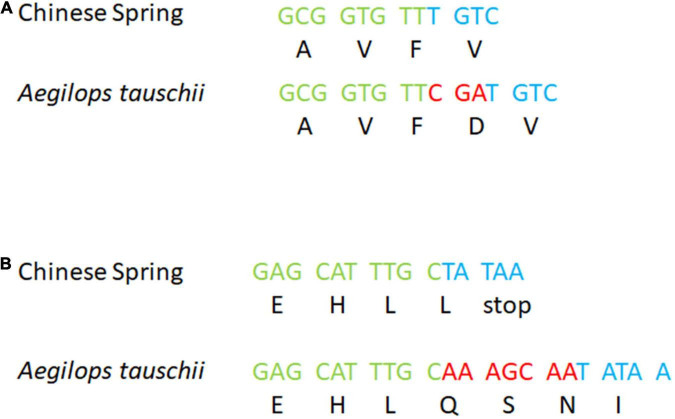
Details of the *Pm2* gene in ‘Chinese Spring’ and *Ae. tauschii*: **(A)** a 3 bp insertion and **(B)** a 7 bp insertion. Respectively, green and blue bases are ‘Chinese Spring’ reference sequences before and after the indel. Red bases are the insertion (found in both *Ae. tauschii* and ‘Maris Huntsman’).

In contrast, the known *Ae. tauschii* introgression into 5DS of the variety ‘Maris Huntsman’ (Wang et al., 2005) was not evidenced by a drop in read coverage. The probe sequences for chromosome 5DS (20,253 sequences) were BLASTed against the assemblies of both ‘Chinese Spring’ and *Ae. tauschii* 5DS resulted in 24,300 hits to the former and 24,173 hits to the latter. The number of probe sequences that had a hit was 20,082 and 19,872, respectively. Those with a single hit were 17,550 and 17,358, respectively (Figure 4A). The percentage similarity between probe sequences and their target was 99.1 and 98.9%, respectively (Figure 4B). Thus, it would appear, the sequences of wheat and *Ae. tauschii* are sufficiently similar over this region that capture probes are equally efficient at capturing sequences from them. To confirm this hypothesis, the sequences surrounding *Pm2*, were compared. Based on the alignment, the ‘Chinese Spring’ and *Ae. tauschii* reference assemblies were highly similar across the 2 Mb of sequence centered on the *Pm2* gene (99.1% similarity); in each, there were 21 annotated genes and synteny appears to be maintained apart from the presence of an inverted repeat of TraesCS5D02G044500 (position 43,382,967–43,386,355) to the upstream position 42,989015–42,992,511 – TraesCS5D02G043600 (Supplementary Table 1). The sequences from ‘Maris Huntsman’ also aligned well to both assemblies. However, within the coding sequence of the *Pm2* gene itself, two indels, one particularly relevant, supported the hypothesis that ‘Maris Huntsman’ is more similar to *Ae. tauschii* than to ‘Chinese Spring’. That is, relative to ‘Chinese Spring’, both *Ae. tauschii* and ‘Maris Huntsman’ carry a 3 bp insertion at position 43,405,954 and a 7 bp insertion at position 43,407,045 (Figure 5^1^).

### Efficacy of Alignment to the Reference Assembly

To further investigate the role of sequence alignment in the regions of reduced sequence coverage, a BLAST search was performed using the mapped and unmapped reads from ‘Bacanora’ against a database containing both *T. aestivum* and *S. cereale* sequences. Of the 1,959 unmapped reads, 709 (36.2%) hit sequences in the BLAST database: 654 (33.4%) to the *S. cereale* 1R sequence and 55 (2.8%) to the *T. aestivum* 1B sequence. Conversely, for the 1,421 reads that had successfully mapped to the *T. aestivum* ‘Chinese Spring’ reference sequence, there were only 167 (11.8%) hits to the *S. cereale* 1R sequence while 1,242 (87.4%) hits to the wheat 1B reference sequence.

**FIGURE 6:**
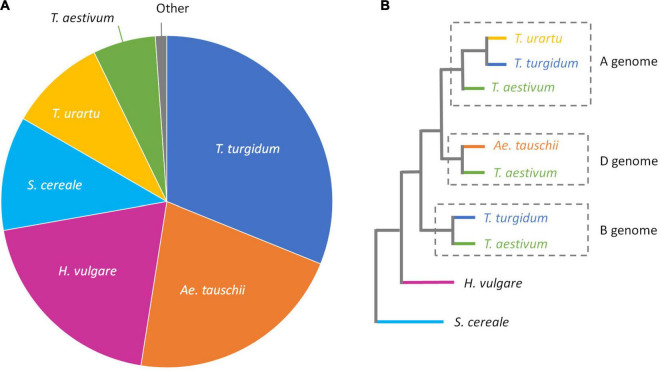
**(A)** Pie chart showing the best BLAST hits against a combined *Poaceae*/*S. cereale* database for captured reads that didn’t map to the IWGSC ‘Chinese Spring’ assembly v1. **(B)** Phylogenetic tree (redrawn from Zhou et al., 2017), showing the relationship of the species used in our *Poaceae*/*S. cereale* database.

For unknown introgressions, it is not possible to compare the unmapped reads to the source sequence. To better understand from where these reads came, an assembly of unmapped reads for all 12 accessions was created and then compared with a database of *Poaceae*/*S. cereale* protein sequences (Figure 6). The unmapped sequences were predominantly (62.1%) found in the progenitor accessions *Triticum turgidum* (AABB genome), *Ae. tauschii* (DD), and *Triticum urartu* (AA). There were also additional hits to the more distant relatives *Hordeum vulgare* (HH) and *S. cereale* (RR).

## Discussion

### Exome Capture

The ‘Gene Capture v1’ and ‘Promoter Capture v1’ probes are based on sequences not only from *T. aestivum* but also *Ae. tauschii* and *T. turgidum* and, thus, should capture sequence from bread wheat and its progenitors (Gardiner et al., 2019). In this study, the exome capture protocol proved effective at capturing a representative genome sample from each of the 12 accessions examined with sequence coverage in distal regions of chromosomes being greater than that across centromeres (Figure 1A); this pattern reflects capture probe distribution which itself is determined by the known telomere to centromere gene gradient (Pingault et al., 2015; Gardiner et al., 2019). Probes appear to have successfully captured wheat sequence and that of introgressions from progenitors as demonstrated by the capture of sequence from the 5DS, *Ae. tauschii* introgression in ‘Maris Huntsman’ (Supplementary Table 1). A review of the literature reporting primary genepool introgression into bread wheat, further indicated that probes were effectively capturing sequence from these introgressions and, thus, resulting in even sequence coverage across such introgressions and the host sequences flanking them. For example, an introgression from *T. turgidum* subsp. *carthlicum* has been reported on 2AL of ‘Renan’ and ‘Riband’ (Chantret et al., 1999; United Kingdom Cereal Pathogen Virulence Survey [UKCVS], 2004); we saw no decrease in sequence coverage for either accession indicating successful capture and alignment. Importantly, this was not just the case for the primary relatives (*T. turgidum* and *Ae. tauschii*) that had been included in the design of the capture probes. An introgression from the primary relative, *Triticum monococcum*, has been reported to be present in 5AL of ‘Maris Huntsman’ (Chen et al., 2021; Supplementary File 1), a *Triticum spelta* introgression has been reported in 2BL of ‘Cadenza’ (Marchal et al., 2018; Supplementary File 1) and introgression from *Triticum dicoccum* has been reported in 3BS of ‘Pavon 76’ (Mago et al., 2014; Supplementary File 1) and none had a corresponding decrease in coverage suggesting adequate capture of these sequences.

The region associated with the *Pm2* gene in ‘Maris Huntsman’ was used as a case study to confirm that sequence diversity present in regions with successful capture and alignment were, indeed, from a wild relative source. Alignment of the captured sequences from ‘Maris Huntsman’ to both the ‘Chinese Spring’ *T. aestivum* reference (IWGSC v1.0) and *Ae. tauschii* (*Ae. tauschii* v4.0 GCF_002575655.1) assemblies showed them to be highly similar. However, two small insertions, with respect to ‘Chinese Spring’, in ‘Maris Huntsman’ and *Ae. tauschii* give support to the hypothesis that ‘Maris Huntsman’ harbors an *Ae. tauschii* introgression (Figure 5). The successful capture of this region is hardly surprising considering that *Ae. tauschii* sequence was used to guide capture probe design (Gardiner et al., 2019) and given the high degree of similarity between the two species, *T. aestivum* and *Ae. tauschii*, *across* the *Pm2* region. Indeed, capture probes designed exclusively from bread wheat sequence may well have proved equally efficacious at capturing sequence from this introgressed region.

The design of the probes, then, has allowed the capture of sequences beyond those belonging exclusively to *T. aestivum*. However, one must expect that beyond a certain level of sequence diversity, a reflection of the evolutionary distance of donors of introgressed segments, probes will no longer capture sequence. Such wide introgressions will not be captured, and coverage of the target will drop. This is a serious limitation if novel regions from more distant relatives are the aim of the capture sequencing and other sequencing methods will need to be employed.

### Alignment to the Reference Assembly

In addition to successful capture and sequencing, one must be able to realign the sequence to the reference (in this case ‘Chinese Spring’ IWGSC v1) for it to be identified as present. There is the potential for the mapping parameters to under-utilize the available sequence as the stringency of the parameters used to align the captured sequences to the ‘Chinese Spring’ reference genome result in some successfully captured sequences being unable to align. Not all variation present in sequencing data is a true reflection of the sequence present and as the alignment stringency is relaxed, sequencing errors may enter the data. To preserve the high-quality sequences, it seems inevitable that diverse sequences will be lost by data processing.

Some mapping protocols, such as the mapping of non-unique hits, can allow for homoeologous sequences to mask gaps in coverage due to deletions or introgressions. In addition, as the mapping of zero in read coverage is not a standard protocol, the gaps seen as a result of diverse sequences are not made apparent (Supplementary Figure 1) and the inability to align diverse sequences to the reference is not reported.

### Efficacy of Alignment to the Reference Assembly

For all 12 accessions, the captured sequences that could not be mapped to the reference were BLASTed against a *Poaceae*/*S. cereale* protein database (Figure 6). Of the sequences that had a hit to the protein database, 62.1% had a match to a sequence derived from a progenitor species (Figure 6). This indicates that some sequences were captured and sequenced but had no corresponding sequence in the ‘Chinese Spring’ reference. Given an alternative reference, some of these sequences may have aligned. The failure of almost 40% of the captured sequences that did not map to the reference probably reflects the limitations of the created *Poaceae*/*S. cereale* protein database since we recognize that there is limited sequence data available for many wheat relatives; the major crop species *T. aestivum*, *T. turgidum*, and *H. vulgare* are well represented in nucleotide databases, but this is not the case for wild relatives. Indeed, we chose to compare our un-mapped sequences to a protein database, rather than a nucleotide database, to maximize the amount of sequence data available. The *Poaceae*/*S. cereale* protein database contained 472,031 sequences. Through this approach, we were able to identify sequences potentially originating from secondary and tertiary genepool species. However, some sequences remained completely unidentified emphasizing that, probably, some diversity is regularly omitted from standard sequencing and alignment. As such, exome capture followed by alignment to a hexaploid reference is not a reliable tool for the identification of introgressions within hexaploid wheat. Where exome capture has been performed and an introgression is suspected, identification is limited by the current availability of wheat relative sequences.

Diverse sequences, such as the *Ae. tauschii* introgression, described in ‘Maris Huntsman’ were successfully captured, sequenced, and aligned in part due to the presence of *Ae. tauschii* sequences in the capture probe set and in part due to the similarity of the progenitor sequence to the D genome of the reference assembly. For the more distant wild relatives, both capture and alignment were less successful. The reduction in mapped sequences was most pronounced in the accessions containing the 1RS.1BL translocation (Figure 1). This is a known introgression that is from a tertiary source. When the 1BS capture probe sequences (26,985) were BLASTed against 1RS of the rye genome assembly (JADQCU000000000 v1), 31% had a hit (Figure 4A), suggesting that some capture would occur, but the percentage similarity between probe sequence and its target was lower in rye than in wheat, suggesting that it might not map back to the reference. This *in silico* assessment was reflected in the captured but un-mapped sequences. By performing a BLAST search against a *T. aestivum* and *S. cereale* database, a number of the unmapped reads in the 1RS containing accession ‘Bacanora’ were found to have matches to the *S. cereale* sequences (33.4%), considerably higher than the *S. cereale* sequences found within the mapped reads of the same accession (11.8%). This suggests that some of the unmapped reads were from regions of 1RS.1BL that were successfully captured but could not be successfully mapped back to the reference. As *S. cereale* sequences are poorly represented in the BLAST database (there were 25,214 out of 472,031 in total), the full extent of *S. cereale* sequences captured is not known and the ratio present may be higher. While it seems that this tertiary relative introgression was not captured to the same extent as a primary genepool relative, it is important to note that some sequences were captured despite the dissimilarity between *S. cereale* and the *T. aestivum* target but the presence of the sequenced further limited by alignment to the reference.

Each of the 12 accessions used in this study, showed reduced read coverage across some regions of at least one of its chromosomes. Most of these drops in coverage were common to several of the accessions studied and, in many cases, they co-located with documented introgressions or with regions where genotyping data had highlighted extensive variability. The accessions ‘Renan’ and ‘Boregar’ had reduced coverage at the end of the short arm of chromosome 2A corresponding to the known introgression from *Ae. ventricosa* associated with rust resistance (*Lr37*, Hanzalová et al., 2007; *Yr17* Dedryver et al., 2009). The size of this introgression has been reported to be c. 33 Mb (Gao et al., 2021) which corresponds with the size of the decline in coverage observed in this study. The eyespot resistance gene, *Pch1* located on the distal end of 7DL, also introduced from *Ae. ventricosa* (Leonard et al., 2007) corresponded to the terminal drop in coverage seen in ‘Boregar’ and ‘Renan’, both reported containing the *Pch1* gene (Burt and Nicholson, 2011). The powdery mildew resistance gene *Pm6* from *T. timopheevii* on 2BL was reported in both ‘Riband’ and ‘Maris Huntsman’ (United Kingdom Cereal Pathogen Virulence Survey [UKCVS], 1996; Wang et al., 2005) and reveals itself as a distinct decrease in coverage in both accessions. Interestingly, this dip is also found in ‘Boregar’ which hasn’t been reported to carry the 2BL introgression but, on the basis of evidence here, probably does. The presence of unreported introgressions is thought to be quite common. For example, several accessions (‘Bacanora’, ‘Boregar’, ‘Cadenza’, ‘KWS Kielder’, ‘Maris Huntsman’, ‘Renan’, and ‘Riband’) shared a large region (c. 45 Mb) with reduced read coverage, which we assume might indicate an introgression, but for which we could find no documentary evidence. This region spans over 640 genes with a range of functions, such as ion channel regulation, phosphorylation, and electron transfer (Supplementary File 4).

Here we demonstrate that there is a relationship between drops in sequence coverage and sequence similarity of the introgression sequence to the region it replaced. That is, introgressions from primary relatives, such as *Ae. tauschii* or *T. dicoccum* (Table 2), are unlikely to fail capture and thus be sequenced and aligned. On the other hand, introgressions from secondary and tertiary genepool species, such as *S. cereale*, *Ae. ventricosa*, and *T. timopheevii*, are likely to avoid capture (Figure 4) and, if captured, fail to align to the reference (Figure 6); such failures are characterized by reduced sequence coverage across the introgressions. The degree of sequence similarity between a wheat relative sequence and the *T. aestivum* equivalent reflects the evolutionary distance. The observations of this study agree with the study in which human exome capture probes were used to capture exome sequences in non-human primates; “*specificity of the capture decreased as evolutionary divergence from humans increase*” (Jin et al., 2012). Exome capture probes designed for *T. aestivum* efficiently captured genic sequences from the D genome progenitor species, *Ae. tauschii*, but performed much less well against *S. cereale*, an evolutionary more distant species belonging to the tertiary genome.

Modern elite wheat varieties carry numerous introgressions which provide genes of important agronomic traits (Table 2), but exome capture may limit the ability to sequence these novel and interesting regions. Introgressions from the primary genepool were successfully captured. Those from more distantly related species, members of the secondary and tertiary genepool, however, were poorly represented in the mapped sequences data (Table 2). While there was evidence that some sequences from secondary and tertiary genepool relatives were present amongst the captured sequences (Figure 6) their number was small and did not map to the reference. Localized reduction in sequence coverage was observed in all 12 accessions studied, including the landrace accessions. Many of these regions of low coverage were collocated with documented introgressions or deletions, while others remain unknown. The method of sequencing used here has essentially limited the diversity of sequence that could be reported. The careful design of capture probes is critically important as lack of capture probe diversity will lead to failure to capture sequence introgressed from distantly related species. The reference genome used will also strongly bias the sequences that can be aligned and so reported as present.

## Experimental Procedures

### Sample Preparation and Sequencing

Genomic DNA from 12 wheat accessions (14 days after germination) was extracted, RNase treated, and purified as described in Burridge et al. (2017).

Individual aliquots in a total volume of 55 μl were sheared to an average of 300 bp using an E220 Focused-ultrasonicator (Covaris, Woburn, MA, United States). SeqCap EZ HyperCap Workflow User’s Guide (Version 2.0) was used with the following modifications. The starting material was increased to 2 μg DNA. The A-tailing reaction was changed to 20°C for 30 min, followed by 65°C for 30 min. Size selection of the pre-capture libraries was replaced with a 0.9 bead: sample ratio. The precapture amplification was changed to nine cycles followed by immediate clean-up. COT human DNA was replaced with 1 μl of Developer Reagent Plant Capture Enhancer (NimbleGen) per 100 ng of DNA.

Exome capture was performed using ‘Gene Capture v1, 4000026820’ and ‘Promoter Capture v1, 4000030160’ wheat capture probes (Gardiner et al., 2019). Gene and Promoter capture probes were not lyophilized but capture reactions performed separately and products combined after post-capture amplification. For the capture wash, the first Wash Buffer I and both Stringent Wash Buffer steps used buffer preheated to 57°C. Fragment size distribution throughout was determined by TapeStation (Agilent) analysis.

Capture probe enriched sequencing libraries were sequenced at the Bristol Genomics Facility using NextSeq 500 and NextSeq500 2 × 150 bp High−Output v2 kit (Illumina). A final library concentration of 0.8 pM was used with a 5% PhiX control library. The full library preparation and capture method are described in detail in Supplementary File 3. All reads are available from the NCBI sequencing read archive using project ID: PRJNA789931.

### Data Analysis

Fastq files for each wheat variety were subjected to quality control using FastQC1 (Babraham Bioinformatics, 2020) and were pre-processed using Fastp (Chen et al., 2018) to trim adaptor sequence and for quality filtering. Paired-end reads were aligned to the ‘Chinese Spring’ reference sequence (IWGSV v1.0) using Burrow-Wheeler Aligner (BWA) (Li and Durbin, 2009) (version 0.7.7-r441), and uniquely mapped reads were identified using sambamba (Tarasov et al., 2015) (version v0.4.4).

Coverage for each chromosome was calculated using samtools (Li et al., 2009) (version 0.1.19-44428cd) using the depth option. Custom perl scripts (available on request) were used to calculate the average depth of coverage for 5 million base pair bins across each chromosome and exome coverage graphs were generated using R (version 3.2.5) (R Core Team, 2013).

Capture probe coverage diagrams were generated with the R package chromPlot using unique location hits and including 0 reads (Verdugo and Oróstica, 2016).

All unmapped reads for the ‘Bacanora’ were extracted from the bam file using samtools (Version: 1.10-24-g383a31b), along with all reads that mapped to the chromosome 1B IWGSC v1.0 reference from physical mapping positions 1–230,000,000 bp (spanning the putative 1B/1RS introgression. These unmapped and mapped reads were then separately queried against a local BLAST database that contained the wheat 1B sequence and the *S. cereale* 1R sequence, using default BLASTN parameters. The top BLAST hit was then parsed from the BLAST output files using custom perl scripts.

Several gnome assemblies were required for this study: IWGSC v1 Chinese Spring assembly; Rye assembly of the Chinese rye cultivar Weining (Li et al., 2021); *Ae. tauschii* subsp. *strangulata* (Luo et al., 2017).

### Exome Capture Probes to 1BS and 5DS

The browser extensible data (BED) file containing the genomic coordinates of the gene capture probes, Wheat_gene_capture_probes.bed, from Gardiner et al. (2019) was downloaded from the Grassroots Data Repository.^2^ From this file, the coordinates for the TGAC v1 probes to chromosomes 1BS and 5DS were extracted. Using the python package pysam, the sequences for these probes were extracted from the TGAC version 1 genome assembly of ‘Chinese Spring’ (Triticum_aestivum.TGACv1.30.dna.genome.fa). The gene capture probe sequences for chromosome 1BS were BLASTed against the chromosome 1BS sequence from the IWGSC v1 assembly and to the chromosome 1RS sequence of the genome assembly of the cultivar ‘Weining’ rye (JADQCU000000000 v1), an elite Chinese *S. cereale* variety (Li et al., 2021). Likewise, the capture probe sequences for chromosome 5DS were BLASTed against the chromosome 5DS sequence from the IWGSC v1 assembly and to the chromosome 5DS from *Ae. tauschii* subsp. *strangulata* assembly, Aet v4.0 (GCA_002575655.1).

### Gene Sequences Surrounding *Pm2* Gene

The putative 5D introgression in ‘Maris Huntsman’ containing the powdery mildew resistance gene *Pm2*, was used as the point of reference. The *Pm2* gene (TraesCS5D02G044600.1) sequence downloaded from EnsemblPlants is 1,266 bp long and produces a protein of 421 aa. To obtain the *Ae. tauschii* homolog, the ‘Chinese Spring’ *Pm2* sequence was BLASTed against the NCBI Triticeae database; the top hit, with 99.3% identity (1,255/1,264), was the *Ae. tauschii* subsp. *strangulata* sequence on 5D (sequence id MW538911.1). The full length of this sequence was 4,421 bp.

To compare sequence similarity of ‘Chinese Spring’ and *Ae. tauschii* coding sequences around the *Pm2* gene, we identified, using the gff3 file for IWGSC v1 (Ensembl Plants genome browser), all the annotated genes within 1 Mb up- and down-stream; in ‘Chinese Spring’, 21 genes were present within this interval (Supplementary Table 1). The sequences of these 21 genes were BLASTed against the NCBI Triticeae database to obtain their homologs in *Ae. tauschii*. These were then BLASTED against the *Ae. tauschii* v 4.0 (GCF_002575655.1) assembly to find their positions.

Using BWA, (Li and Durbin, 2009) we aligned the ‘Maris Huntsman’ captured sequences against both ‘Chinese Spring’ (IWGSC v1.0) and *Ae. tauschii* (Aet V4.0) assemblies. Both assemblies and the ‘Maris Huntsman’ BAM files were indexed using Samtools. The gff3 file of the ‘Chinese Spring’ assembly was also downloaded. An equivalent gff3 file for *Ae. tauschii* was created based on the positions obtained by BLAST and the regions viewed in IGV (Robinson et al., 2011).

The ‘Maris Huntsman’ captured sequences were aligned, using BWA, to both CS and Aet the sequences around the *Pm2* gene (TraesCS5D02G044600 in ‘Chinese Spring’ and AET5Gv20114600 in *Ae. tauschii*) in the accession ‘Maris Huntsman’ to that of the ‘Chinese Spring’ assembly (IWGSC v1.0) and *Ae. tauschii* v4.0. For both assemblies, using pysam, pulled out the sequence for the *Pm2* gene (c. 4,420 bp) plus 1 Mb both up and downstream from it. In both assemblies, this region contains 21 genes.

We were interested to see whether the exome captured sequences from ‘Maris Huntsman’ 5DS had greater similarity to the gene sequences of ‘Chinese Spring’ or those of *Ae. tauschii*. Because we believed that the putative introgression contained the powdery mildew resistance gene *Pm2*, we used this gene as our point of reference. We began by pulling down the *Pm2* gene (TraesCS5D02G044600.1) sequence from EnsemblPlants; this is 1,266 bp long and produces a protein of 421 aa. To obtain the homolog from *Ae. tauschii*, we BLASTed the ‘Chinese Spring’ *Pm2* sequence (TraesCS5D02G044600) against the NCBI Triticeae database; the top hit was the homologous gene on 5D of *Ae. tauschii* subsp. *strangulata* (AET5Gv20114600). This sequence was then BLASTed against the NCBI Triticeae database. With 99.3% identity (1,255/1,264), it hit the *Ae. tauschii Pm2* sequence (MW538911.1), which has a full-length functional gene of 4,421 bp.

## Data Availability Statement

The datasets presented in this study can be found in online repositories. The names of the repository/repositories and accession number(s) can be found below: https://www.ncbi.nlm.nih.gov/, PRJNA789931. The SNP markers and genotypes are available from the CerealsDB website (Wilkinson et al., 2016): https://www.cerealsdb.uk.net/cerealgenomics/CerealsDB/array_info.php.

## Author Contributions

AB prepared the samples and took the lead in writing the manuscript. MW carried out the computational analyses and contributed to the manuscript. PW and GB carried out the computational analyses. KE and GB secured the required funding and supervised the project. All authors helped to interpret the data and contributed to the final manuscript.

## Conflict of Interest

The authors declare that the research was conducted in the absence of any commercial or financial relationships that could be construed as a potential conflict of interest.

## Publisher’s Note

All claims expressed in this article are solely those of the authors and do not necessarily represent those of their affiliated organizations, or those of the publisher, the editors and the reviewers. Any product that may be evaluated in this article, or claim that may be made by its manufacturer, is not guaranteed or endorsed by the publisher.

## Footnotes

1
https://plants.ensembl.org/Triticum_aestivum/Location/Compara_Alignments?align=9814–Aegilops_tauschii–5D:46999777-47002088;db=core;g=TraesCS5D02G044600;r=5D:43405783-43407148;t=TraesCS5D02G044600.1

2
https://opendata.earlham.ac.uk/wheat/under_license/toronto/Gardiner_2018-07-04_Wheat-gene-promoter-capture/

## Supplementary Material

Supplementary Figure 1 Sequence coverage diagrams for chromosome 2B of the accession ‘Riband’ using different alignment parameters. **(A)** Average depth of coverage across 5 Mb bins using any good hit to the 2B reference. **(B)** Average depth of coverage across 5 Mb bins using only sequences that give a unique hit to the 2B reference and allowing the display of zero reads.
