# Supplementary figures and images for: The Use and Limitations of Exome Capture to Detect Novel Variation in the Hexaploid Wheat Genome
Source: Front Plant Sci. 2022 Apr 12;13:841855. doi: 10.3389/fpls.2022.841855 (PMC9039655; doi:10.3389/fpls.2022.841855)

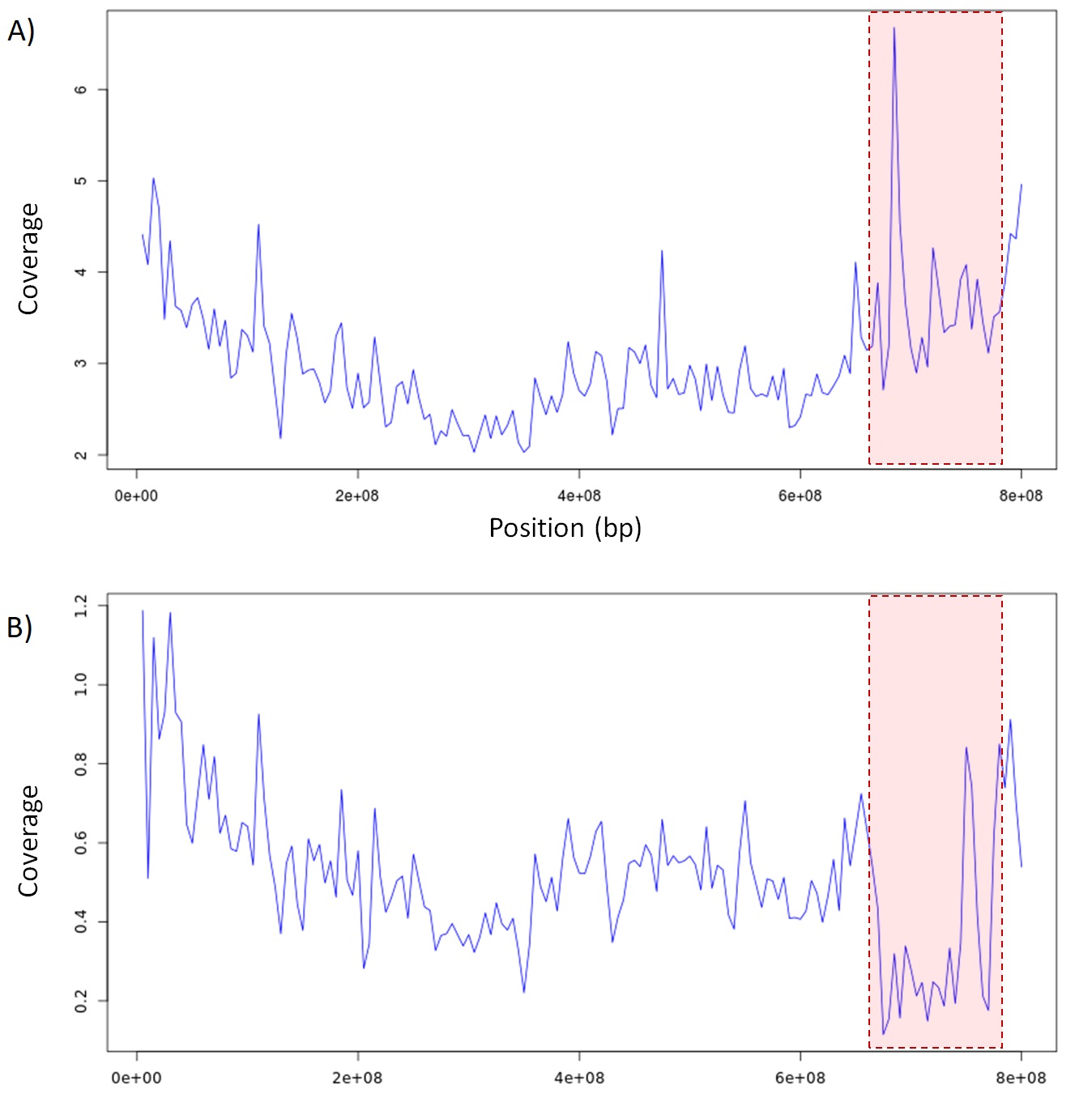

Supplement: Supplementary Figure 1 — Sequence coverage diagrams for chromosome 2B of the accession ‘Riband’ using different alignment parameters. (A) Average depth of coverage across 5 Mb bins using any good hit to the 2B reference. (B) Average depth of coverage across 5 Mb bins using only sequences that give a unique hit to the 2B reference and allowing the display of zero reads. [file Image_1.jpeg]
