# Supplementary material for: The Use and Limitations of Exome Capture to Detect Novel Variation in the Hexaploid Wheat Genome
Source: Front Plant Sci. 2022 Apr 12;13:841855. doi: 10.3389/fpls.2022.841855 (PMC9039655; doi:10.3389/fpls.2022.841855)
Supplement: Supplementary file 2 [file Data_Sheet_1.PDF]

2B

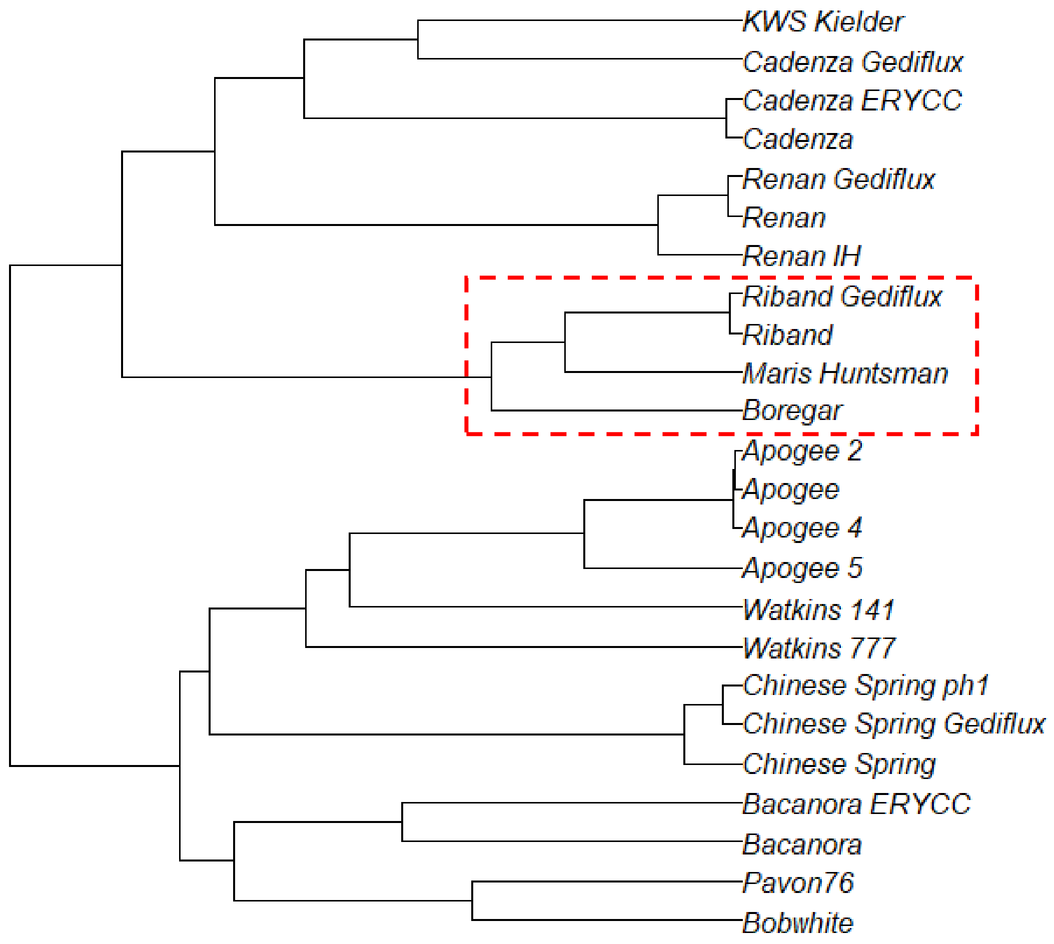

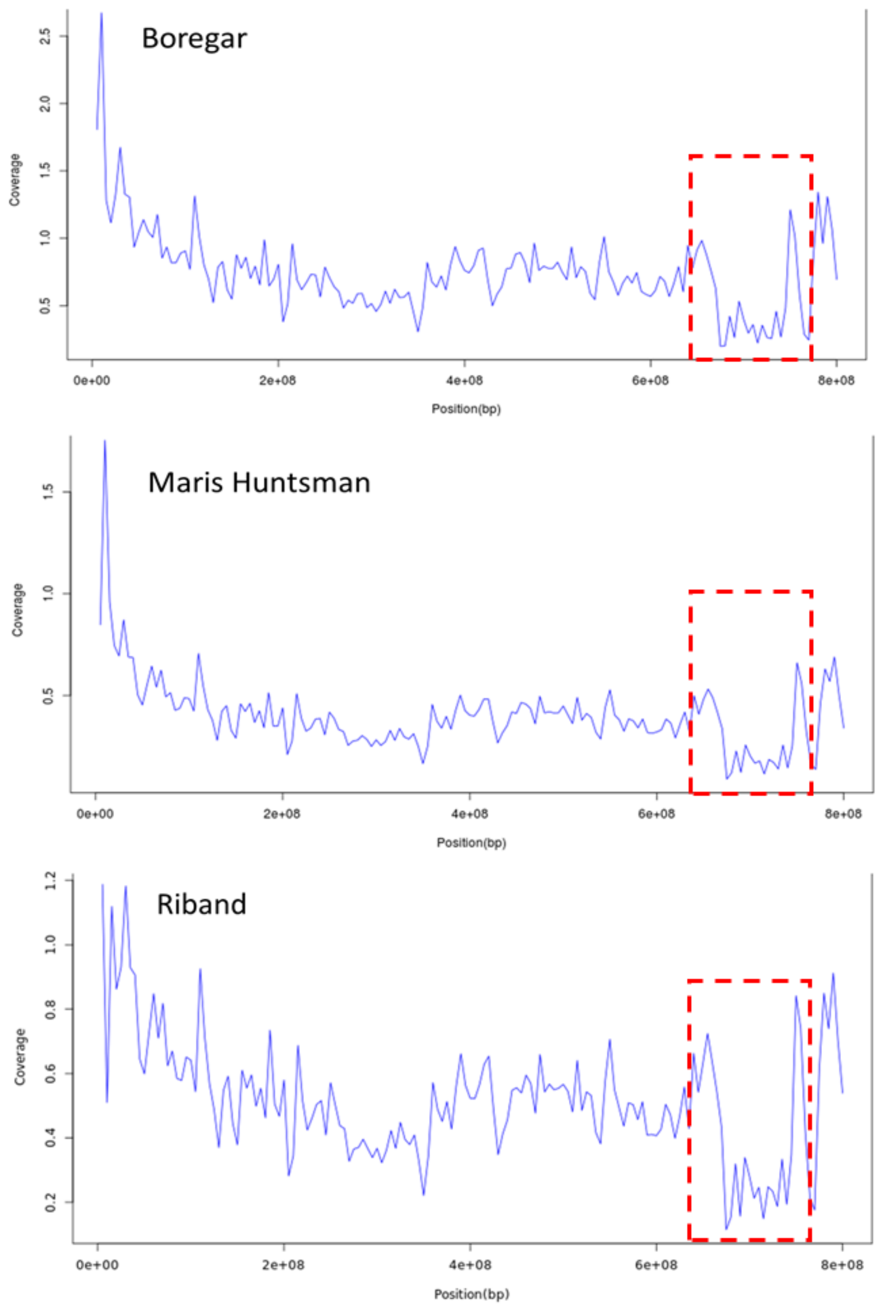

Based on 2083 markers from the 35K Wheat Breeders' Array

2DL

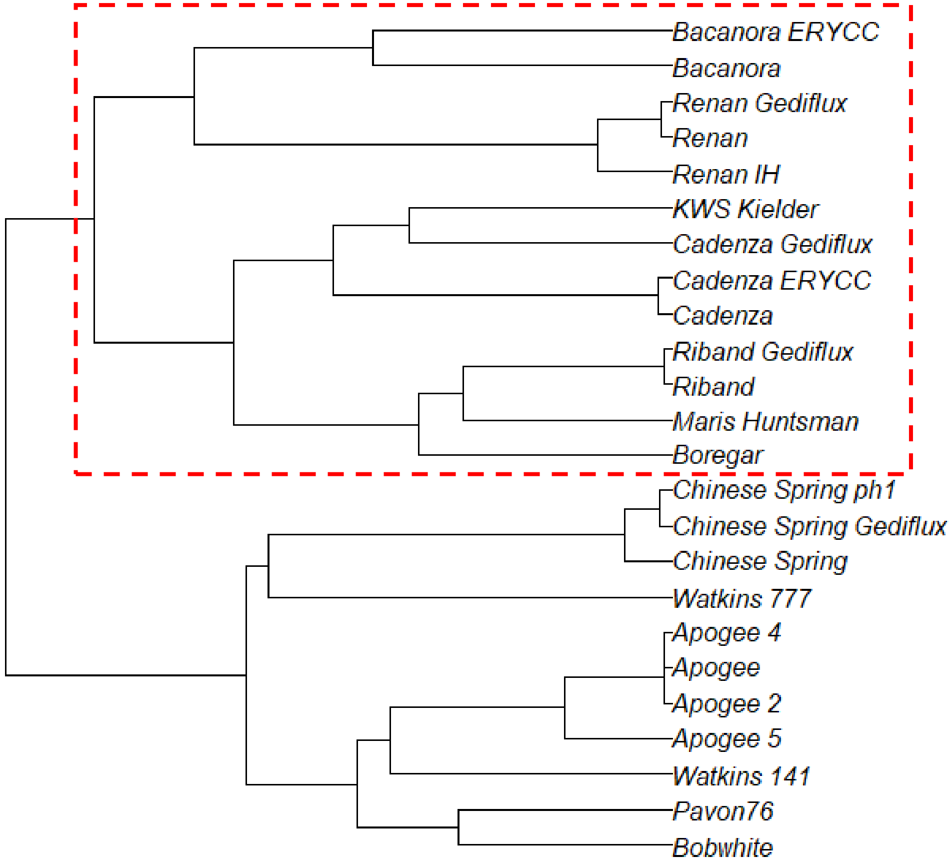

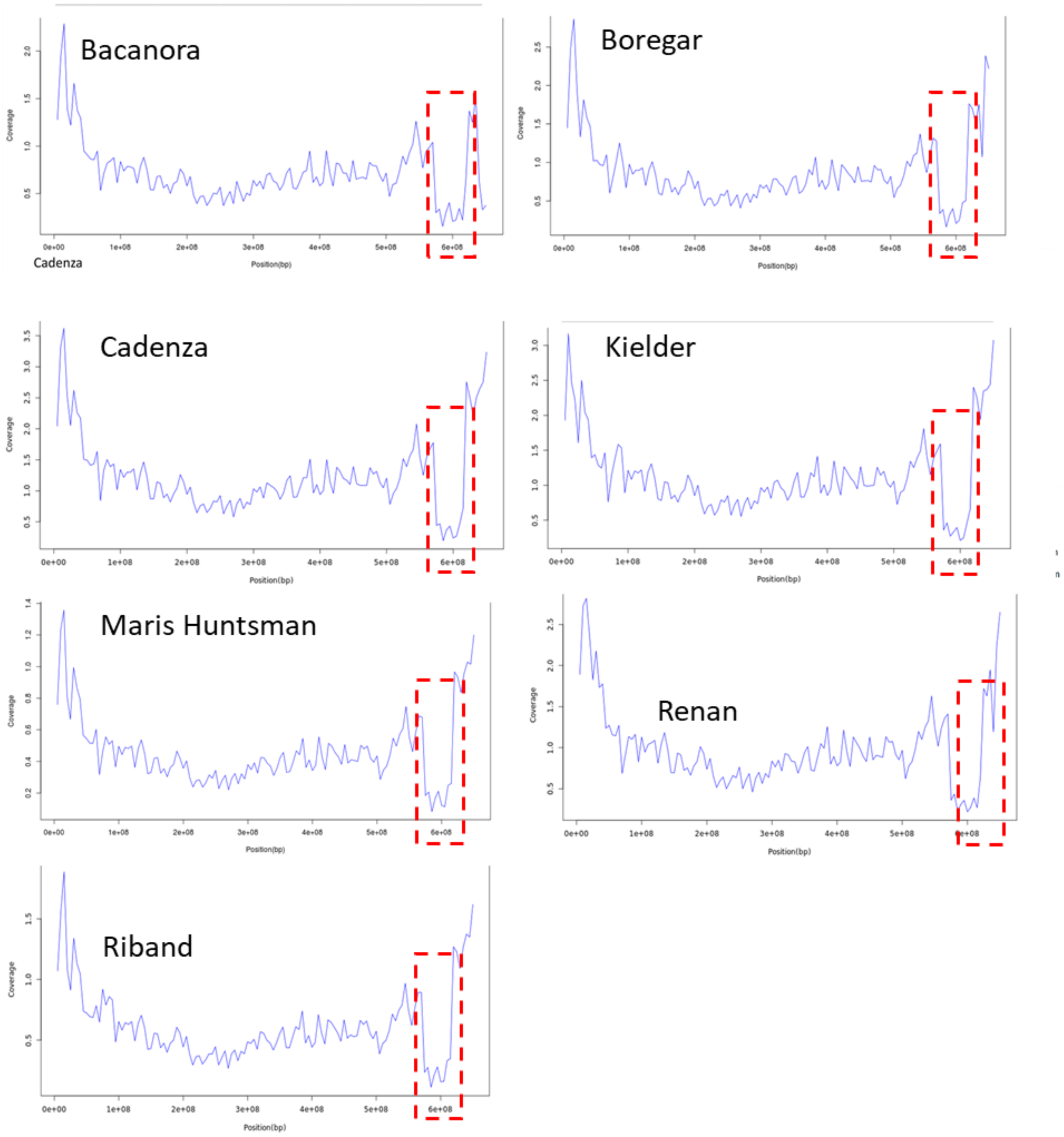

Based on 2237 markers from the 35K Wheat Breeders’ Array
