# Supplementary material for: The Use and Limitations of Exome Capture to Detect Novel Variation in the Hexaploid Wheat Genome
Source: Front Plant Sci. 2022 Apr 12;13:841855. doi: 10.3389/fpls.2022.841855 (PMC9039655; doi:10.3389/fpls.2022.841855)
Supplement: Supplementary file 3 [file Data_Sheet_2.PDF]

The Use and Limitations of Exome Capture to Detect Novel Variation in the Hexaploid Wheat Genome - Supplementary Method

The library preparation uses the KAPA HyperPrep kit (not to be confused with HyperPlus) and follows the Roche Manual 1.2 with some adaptations required due to in-house equipment. The exome capture method is based on the Roche Manual 2.0 with changes suggested in Gardiner, *et al.* (2019), changes to the buffer temperature regime as suggested in personal communication with Karim Gharbi, Leah Catchpole and Thomas Brabbs at the Earlham Institute and some in house optimisations.

1. Prepare the Sample Library using KAPA Hyper Prep Kit

1.1 Shearing

Shearing is performed using the E220 Focused- ultrasonicator (Covaris, MA, US). Pipette 2ug DNA in a final volume of 55ul per sample into microtube model E220_500444 (8 microTUBE-50 Strip V2 -10mm offset)

Peak Power: 75

Duty Factor: 20

Cycles per Burst: 1000

Time: 72s

Average fragment size of 300bp confirmed using TapeStation

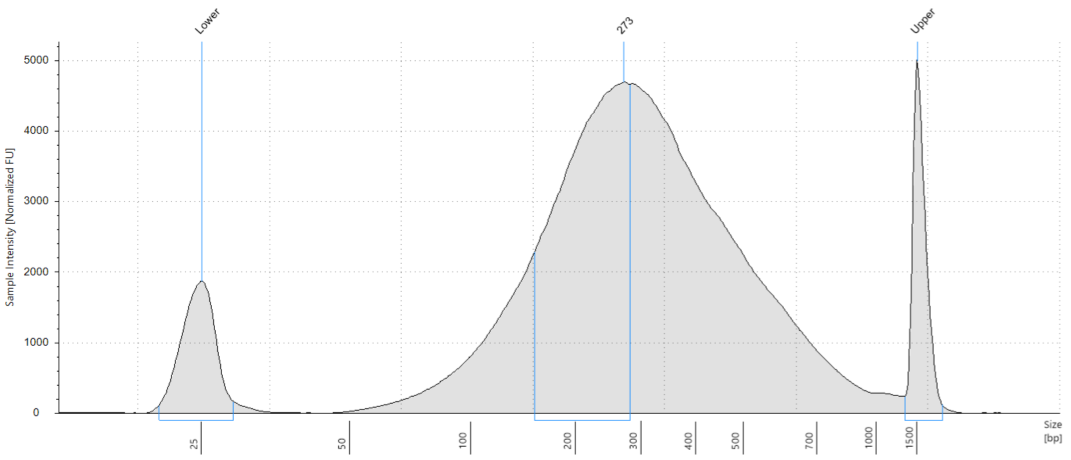

Sheared DNA should be around 300bp, although there will be some variation between samples with the same settings. Times can vary depending on the sonicator model and

consumables used. If fragments are too large, increase the shearing time, if fragments are too small, decrease the shearing time.

1.2 End Repair and A-tailing

1. Prepare a master mix of the following reagents:

	Per Sample
KAPA End Repair & A-Tailing Buffer	7 µL
KAPA End Repair & A-Tailing Enzyme Mix	3 µL

These are contained in the KAPA Hyper Prep Kit (07 962 312 001)

3. Add 10 µL of End Repair and A-tailing Master Mix, to each sample.

4. Mix, centrifuge briefly and immediately place on the thermocycler.

Step 1: 30 minutes at 20 °C

Step 2: 30 minutes at 65 °C

Step 3: Hold at 4 °C

1.3 Adapter Ligation

1. Prepare a master mix of the following reagents:

	Per Sample
PCR-grade water	5 µL
KAPA Ligation Buffer	30 µL
KAPA DNA Ligase	10 µL

These are contained in the KAPA Hyper Prep Kit (07 962 312 001)

2. Add 5ul of the chosen Index to the sample.

Samples used the following index:

Accession	p5	p7
Apogee	TATAGCCT	CGCTCATT
Bacanora	TATAGCCT	GAGATTCC
Bobwhite	AGGCGAAG	TAATGCGC
Boregar	TAATCTTA	TCCGGAGA
Cadenza	AGGCGAAG	AGCGATAG
KWS Kielder	ATAGAGGC	GAGATTCC

Maris Huntsman	ATAGAGGC	ATTCAGAA
Pavon 76	CCTATCCT	GAATTCGT
Renan	CAGGACGT	TCCGCGAA
Riband	GGCTCTGA	CTGAAGCT
Watkins 777	TAATCTTA	CGGCTATG

3. Add 45 µL of the Ligation Master Mix.

4. Mix, centrifuge briefly and immediately incubate at 20 °C for 30 minutes.

1.3 Post-Ligation Cleanup

1. Vortex the room-temperature AMPure reagent and add to the ligation reaction product in a 0.9 ratio.

	Per Sample
Ligation reaction product	110 µL
AMPure XP Reagent	99 µL

Adjust the volumes to the correct ratio if the ligation reaction product volume differs.

2. Vortex thoroughly and incubate the sample at room temperature for 5 minutes.

3. Place on a magnetic particle collector and allow 5 minutes for the liquid to clear.

4. Carefully remove and discard the supernatant, add 200 µL of freshly-prepared 80% ethanol and incubate the sample at room temperature for ≥30 seconds.

5. Carefully remove and discard the ethanol, add 200 µL of freshly-prepared 80% ethanol and incubate the sample at room temperature for ≥30 seconds.

6. Carefully remove and discard the ethanol. Allow the beads to dry at room temperature, sufficiently for all the ethanol to evaporate, but not for the beads to over-dry.

7. Remove the sample from the magnetic particle collector and resuspend in 53 µL of EB-buffer (10 mM Tris-HCl, pH 8.0).

8. Vortex and incubate away from magnetic particle collector for 5 minutes.

9. Place on a magnetic particle collector and allow 5 minutes for the liquid to clear.

14. Transfer 50 µL supernatant to a fresh tube/well.

1.4 Pre-Capture LM-PCR

1. Prepare a master mix of the following reagents:

	Per Sample
KAPA HiFi HotStart ReadyMix (2X)	25 μL
Library Amplification Primer Mix (10X)	5 μL

These are contained in the KAPA Hyper Prep Kit (07 962 312 001)

2. To each sample library add 30 μL of PreCapture LM-PCR Master Mix.

3. Mix, centrifuge briefly and immediately place on the thermocycler

Step 1: 45 seconds at 98 °C	
Step 2: 15 seconds at 98 °C	9 total cycles
Step 3: 30 seconds at 60 °C	
Step 4: 30 seconds at 72 °C	
Step 6: 1 minute at 72 °C	
Step 7: Hold at 4 °C	

1.5 Purify the Amplified Sample

1. Vortex the room-temperature AMPure reagent and add to the amplified sample in a 1.8 ratio.

	Per Sample
Amplified sample	80 μL
AMPure XP Reagent	144 μL

Adjust the volumes to the correct ratio if the amplified sample volume differs.

2. Vortex thoroughly and incubate the sample at room temperature for 5 minutes.

3. Place on a magnetic particle collector and allow 5 minutes for the liquid to clear.

4. Carefully remove and discard the supernatant, add 200 μL of freshly-prepared 80% ethanol and incubate the sample at room temperature for ≥30 seconds.

5. Carefully remove and discard the ethanol, add 200 μL of freshly-prepared 80% ethanol and incubate the sample at room temperature for ≥30 seconds.

6. Carefully remove and discard the ethanol. Allow the beads to dry at room temperature, sufficiently for all the ethanol to evaporate, but not for the beads to over-dry.

7. Remove the sample from the magnetic particle collector and resuspend in 53 μL of PCR-grade water.

8. Vortex and incubate away from magnetic particle collector for 5 minutes.

9. Place on a magnetic particle collector and allow 5 minutes for the liquid to clear.

14. Transfer 50 µL supernatant to a fresh tube/well.

Use the TapeStation at this stage to check both quality and quantity of library.

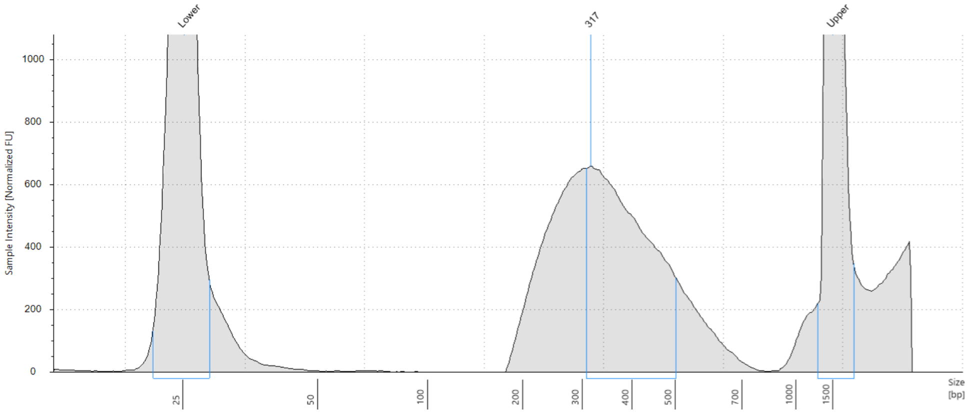

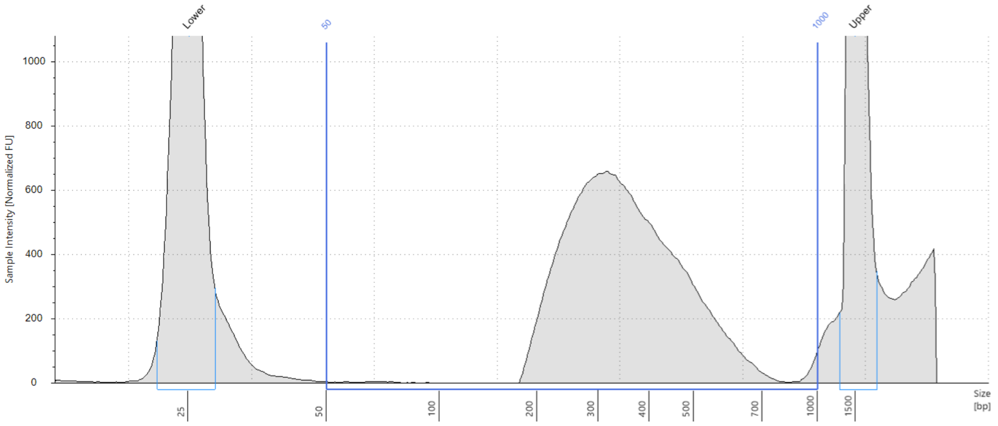

The library above 190bp has been completely removed with a new peak of 317 (original 300bp fragments plus adapters). The additional material above 1000bp should not bind to the capture baits, but they will give a false high concentration if using Qubit or nanodrop.

2. Perform Library Capture and Purification

2.1 Hybridization to Capture Probes

1. Using the TapeStation measurements for the library concentration only, combine all 12 libraries to obtain a single pool with a combined mass of 2 µg. Divide into two 1ug aliquots.

2. Add 10 µL of SeqCap EZ Developer Reagent to each 1 µg sample library pool.

3. Add 7 µL of the HyperCap Universal Blocking Oligos to each sample library pool.

4. Add 2 volumes of room-temperature vortexed AMPure XP Beads to each sample library pool (volumes will vary).

5. Vortex thoroughly and incubate each sample library pool at room temperature for 10 minutes.

6. Place on a magnetic particle collector and allow 5 minutes for the liquid to clear.

7. Carefully remove and discard the supernatant, add 190 µL of freshly-prepared 80% ethanol and incubate the sample at room temperature for ≥30 seconds.

8. Carefully remove and discard the ethanol. Allow the beads to dry at room temperature, sufficiently for all the ethanol to evaporate, but not for the beads to over-dry.

9. Prepare a master mix of the following reagents:

	Per Capture
Hybridization Buffer (2X)	7.5 µL
Hybridization Component A	3 µL

10. Add 10.5 µL of the Hybridization Buffer/Hybridization Component A mix to the beadbound DNA samples.

2. Vortex thoroughly and incubate the sample at room temperature for 5 minutes.

3. Place on a magnetic particle collector and allow 5 minutes for the liquid to clear.

4. Carefully transfer 10.5 µL of supernatant to a fresh tube/well containing 4.5 µL of gene or promoter probe pool. (Gene Capture v1, 4000026820 or Promoter Capture v1, 4000030160)

5. Vortex thoroughly, centrifuge briefly and immediately place on the thermocycler with hotlid set to 57°C.

Step 1: 95 °C for 5 minutes

Step 2 47 °C for 18 hours (1,080 minutes)

Step 3: 47°C Hold

Proceed immediately after 18h

2.2 Reagent Preparation

Prepare and dilute 10X Wash Buffers (I, II, III and Stringent) and 2.5X Bead Wash Buffer:

Buffer	Buffer Stock	Water	1X buffer	Preparation
10X Stringent Wash Buffer (vial 4)*	40 µL	360 µL	400 µL	Pre-heat in thermal cycler

10X Wash Buffer I (vial 1)*	30 µL	270 µL	300 µL	Pre-heat one wash in thermal cycler. Leave remainder at room temperature
10X Wash Buffer II (vial 2)	20 µL	180 µL	200 µL	
10X Wash Buffer III (vial 3)	20 µL	180 µL	200 µL	
2.5X Bead Wash Buffer (vial 7)	100 µL	150 µL	250 µL	

These buffers are contained in the SeqCap Hybridization and Wash Kit * Stock solutions of Stringent Wash Buffer and Wash Buffer I will not dissolve fully at room temperature. Incubate at 35 °C before dilution to ensure stock is fully in solution

2.3 Bead Washing

1. Aliquot 100 µL of room-temperature beads for each capture.

2. Place on a magnetic particle collector and allow 5 minutes for the liquid to clear.

3. Carefully remove and discard the supernatant, add 200 µL of 1X Bead Wash Buffer.

4. Vortex thoroughly, place on a magnetic particle collector and allow 5 minutes for the liquid to clear.

5. Carefully remove and discard the supernatant, add 200 µL of 1X Bead Wash Buffer.

6. Vortex thoroughly, place on a magnetic particle collector and allow 5 minutes for the liquid to clear.

7. Carefully remove and discard the supernatant, add 100 µL of 1X Bead Wash Buffer and vortex thoroughly

8. Aliquot 50 µL of beads into new tube/well for each capture.

9. Place on a magnetic particle collector and allow 5 minutes for the liquid to clear.

10. Carefully remove and discard the supernatant.

Proceed immediately to the next step, do not allow the beads to dry out. Delay the last step if the samples are not yet ready.

2.4 Binding the Samples to the Beads

At the end of the 18h incubation, maintain the temperature.

1. Transfer each hybridization sample to a single prepared tube/well of capture beads and vortex thoroughly.

2. Incubate samples at 47 °C for 45 minutes (heated lid at 57 °C). Pre-heat 1x Wash buffer 1 and 1x Stringent Wash Buffer.

2.5 Wash and Recover

1. Add 100 µL of pre-heated 1X Wash Buffer I to the sample.
2. Vortex thoroughly, place on a magnetic particle collector and allow 5 minutes for the liquid to clear.
3. Carefully remove and discard the supernatant, add 200 µL of pre-heated 1X Stringent Wash Buffer to each sample
4. Vortex thoroughly and incubate at 47 °C, close lid (set to 57 °C) 5 minutes
5. Place on a magnetic particle collector and allow 5 minutes for the liquid to clear.
6. Carefully remove and discard the supernatant, add 200 µL of pre-heated 1X Stringent Wash Buffer to each sample
7. Vortex thoroughly and incubate at 47 °C, close lid (set to 57 °C) 5 minutes
8. Place on a magnetic particle collector and allow 5 minutes for the liquid to clear.
9. Carefully remove and discard the supernatant, add 200 µL of room temperature 1X Wash Buffer 1 to each sample
10. Vortex thoroughly and incubate at room temperature for 1 minute
11. Place on a magnetic particle collector and allow 5 minutes for the liquid to clear.
12. Carefully remove and discard the supernatant, add 200 µL of room temperature 1X Wash Buffer II to each sample
13. Vortex thoroughly and incubate at room temperature for 1 minute
14. Place on a magnetic particle collector and allow 5 minutes for the liquid to clear.
15. Carefully remove and discard the supernatant, add 200 µL of room temperature 1X Wash Buffer III to each sample
16. Vortex thoroughly and incubate at room temperature for 1 minute
17. Place on a magnetic particle collector and allow 5 minutes for the liquid to clear.
18. Carefully remove and discard the supernatant, add 15 µL of PCR-grade water.

2.6 Post Capture Amplification

1. Prepare a master mix of the following reagents:

KAPA HiFi HotStart ReadyMix (2X)	25 µL
Post-LM-PCR Oligos 1 & 2, 5 µM	5 µL

These are in the HyperCapTarget Enrichment Kit.

2. Add 30 µL Post-Capture LM-PCR Master Mix to each bead-bound DNA sample.

3. Mix thoroughly by pipetting and immediately place on the thermocycler with a 57°C hotlid.

Step 1: 45 seconds at 98 °C

Step 2: 15 seconds at 98 °C
Step 3: 30 seconds at 60 °C
Step 4: 30 seconds at 72 °C

Repeat for a total 14 cycles

Step 6: 1 minutes at 72 °C
Step 7: Hold at 4 °C

2.7 Purify the Amplified and Captured Sample

1. Add 90 µL of room-temperature vortexed AMPure XP Beads to the 50 µL amplified captured Multiplex DNA Sample library.

2. Vortex thoroughly and incubate at room temperature for 5 minutes.

3. Place on a magnetic particle collector and allow 5 minutes for the liquid to clear.

4. Carefully remove and discard the supernatant, add 200 µL of freshly-prepared 80% ethanol and incubate the sample at room temperature for ≥30 seconds.

5. Carefully remove and discard the ethanol, add 200 µL of freshly-prepared 80% ethanol and incubate the sample at room temperature for ≥30 seconds.

6. Carefully remove and discard the ethanol. Allow the beads to dry at room temperature, sufficiently for all the ethanol to evaporate, but not for the beads to over-dry.

7. Remove the sample from the magnetic particle collector and resuspend in 53 µL of PCR-grade water.

8. Vortex thoroughly and incubate away from magnetic particle collector for 5 minutes.

9. Place on a magnetic particle collector and allow 5 minutes for the liquid to clear.

14. Transfer 50 µL supernatant to a fresh tube/well.

Use TapeStation to confirm the library concentration prior to sequencing.
